# Supplementary material for: Content-rich biological network constructed by mining PubMed abstracts
Source: BMC Bioinformatics. 2004 Oct 8;5:147. doi: 10.1186/1471-2105-5-147 (PMC528731; doi:10.1186/1471-2105-5-147)
Supplement: Additional File 5 — The original Chilibot query results of the term "long-term potentiation (LTP)" and 22 other terms, limiting the latest references analyzed to the years 1990, 1995, 2000, and 2004. [file 1471-2105-5-147-S5.bz2 › chilibotAdditionalFile5/ltp2000/html/PKC_CAMKII.html]

 


 **PKC** and **CAMKII** 
  
Found 61 abstracts in PubMed,  **30 abstracts were retrieved and analyzed**.  


---

 Search Google  |
 PDF files only 
|  EDU domain only 

---

**Interactive relationship** (e.g. stimulation, inhibition, etc)

- We confirmed the marked translocation of  **PKC**  and  **CaMKII**  to cell membranes induced by ischemia, concomitantly with a decrease in the  **PKC**  activity in both the membrane fraction and cytosol.  Ref: 10592293 Brain Res, 1999
- 2 Polymyxin B PMB, an inhibitor of protein kinase C  **PKC** , markedly suppressed the peptide activation of MAPK, but did not affect  **CaMKII**  autophosphorylation.  Ref: 10375750 Zhongguo Yao Li Xue Bao, 1998
- We report that Wnt and Frizzled homologs that activate calcium release and  **PKC**  also activate  **CamKII**  activity in Xenopus embryos, while Wnt and Frizzled homologs that activate beta catenin function do not.  Ref: 10777564 J Biol Chem, 2000

- :-)

  **Parallel relationship** (e.g. studied together, co-existance, homology, etc.)

  - 3, a putative receptor mediated signaling pathway involving second messenger IP3, immediately early gene c fos transcription and protein kinase  **PKC** ,  **CaMKII**  and MAPK.  Ref: 10074788 Prog Brain Res, 1998
  - In contrast, the protein synthesis inhibitor, cycloheximide, the selective inhibitor of cAMP dependent protein kinase PKA, H 89, the selective inhibitors of protein kinase C  **PKC** , PKCI and GF109203X, the mitogen activated protein MAP kinase kinase inhibitor, PD98059, or the inactive  **CaMKII**  inhibitors, KN 92 and NP218, had no effect on the currents.  Ref: 10101246 Brain Res Mol Brain Res, 1999
  - There were similar total protein and phosphoprotein profiles in the MVN and PH, as well as both  **PKC**  and  **CaMKII**  activity, suggesting that the MVN and PH are similar in the way that proteins undergo rapid modification by phosphorylation.  Ref: 10939686 J Vestib Res, 2000
  - ...  **CaMKII**  281 309 and  **PKC**  19 31 had no effect on the ATP dependent activation.  Ref: 10617655 J Biol Chem, 2000
  - Treatment of Xenopus oocytes with specific inhibitors of  **PKC** , PKA,  **CamKII** , tyrosine kinases, and serine threonine protein phosphatases did not affect the 100 mM ethanol induced inhibition of GluR6 receptor mediated currents.  Ref: 9756045 Alcohol Clin Exp Res, 1998
  - Neither PKI 5 24 nor  **CaMKII**  281 309 or  **PKC**  19 31 affected the ATP dependent activation.  Ref: 10617655 J Biol Chem, 2000
  - Phosphorylation consensus sites for various kinases exist within the sequence of the potassium channel subunit Kv4.2, including consensus sites for extracellular signal regulated kinase mitogen activated protein kinase ERK MAPK, protein kinase A PKA, protein kinase C  **PKC** , and calcium calmodulin dependent kinase II  **CaMKII** , and kinase assays have shown that particular amino acids of the consensus sites are bonafide phosphorylation sites in vitro.  Ref: 11040264 Learn MemLearn Mem, 2000
  - Using a cardiac arrest model of global brain ischemia of 10 min duration, we investigated the effect of hyperglycemia 20 mM and hypercapnia pCO 2 300 mmHg on the subcellular redistribution of  **PKC**  alpha, beta, gamma and  **CaMKII**  to synaptic membranes and to the microsomes, as well as the effect on  **PKC**  activity.  Ref: 10592293 Brain Res, 1999
  - These results support biochemical observations indicating that  **PKC** , similar to calcium calmodulin dependent protein kinase II  [ **CAMKII** ] , has an important role in controlling synaptic strength via modulation of AMPA receptor function, potentially through the direct phosphorylation of the GluR1 subunit.  Ref: 9819284 J Neurophysiol, 1998
  - As calcium release was the first described feature of the noncanonical pathway, and as calcium probably plays a key role in the activation of  **CamKII**  and  **PKC** , we have named this Wnt pathway the Wnt calcium pathway.  Ref: 10858654 Trends Genet, 2000
  - An aberrant cell signaling following ischemia has been proposed to be involved in ischemic cell death, affecting protein kinase C  **PKC**  and the calcium calmodulin kinase II  **CaMKII** .  Ref: 10592293 Brain Res, 1999
  - To illustrate the capabilities of the technique, the activation of protein kinase C  **PKC** , protein kinase A PKA, calcium calmodulin activated kinase II  **CamKII** , and cdc2 protein kinase cdc2K was measured in response to both pharmacological or physiological stimuli.  Ref: 10700147 Nat Biotechnol, 2000
  - These results implicate  **PKC** ... signal transduction pathways in the initiation of SN compensation in guinea pig.  Ref: 11056183 Brain Res, 2000
  - SyntagI, the purified, recombinant, cytoplasmic domain of rat synaptotagmin I, was an effective substrate in vitro for calcium calmodulin dependent protein kinase II  **CaMKII** , protein kinase C  **PKC** , and casein kinase II caskII.  Ref: 10461881 J Neurochem, 1999
  - The cyclic changes of fEPSPs were blocked by inhibitors of protein kinase C  **PKC**  but were unaffected by inhibitors of calcium calmodulin dependent protein kinase II  **CaMKII**  or myosin light chain kinase MLCK.  Ref: 9527618 Neurosci Res, 1997
  - Previous studies demonstrated that Ser655 of APP was phosphorylated by protein kinase C  **PKC**  and calmodulin dependent protein kinase II  **CaMKII**  in vitro and by unidentified protein kinase s in vivo.  Ref: 10329382 Biochem Biophys Res Commun, 1999
  - In contrast, LTM appears to require PKA and  **PKC**  activity in both structures, guanylyl cyclase, PKG, and  **CaMKII**  activity in CA1, and MAPKK activity in the entorhinal cortex.  Ref: 10704324 Neurobiol Learn Mem, 2000
  - When the NMDA channels are open, the determinant role in receptor phosphorylation is played by the  **PKC**  and  **CaMKII** .  Ref: 10885014 Ross Fiziol Zh Im I M Sechenova, 2000
  - By contrast other Wnts stimulate intracellular calcium release and activate two kinases,  **CamKII**  and  **PKC** , in a G protein dependent manner.  Ref: 10858654 Trends Genet, 2000
  - The drugs and doses were the guanylyl cyclase inhibitor LY83583 LY, 2.5 microMg, the inhibitor of Tyr protein kinase at low concentrations and of protein kinase G PKG at higher concentrations lavendustin A LAV, 0.1 and 0.5 microMg, the PKG inhibitor KT5823 2.0 microMg, the protein kinase C  **PKC**  inhibitor staurosporin STAU, 2.5 microMg, the inhibitor of calcium calmodulin protein kinase II  **CaMKII**  KN62 3.6 microMg, the protein kinase A PKA inhibitor KT5720 0.5 microMg, and the mitogen activated protein kinase kinase MAPKK inhibitor PD098059 PD, 0.05 microMg.  Ref: 10704324 Neurobiol Learn Mem, 2000
  - Using phosphorylation site specific antibodies, we found that induction of chem LTD produces a persistent dephosphorylation of the GluR1 subunit of AMPA receptors at serine 845, a cAMP dependent protein kinase PKA substrate... a substrate of protein kinase C  **PKC**  and calcium calmodulin dependent protein kinase II  [ **CAMKII** ]   **CaMKII** .  Ref: 9856470 Neuron, 1998
  - The results suggest the possibility that the phosphorylation of synaptotagmin I by  **CaMKII**  and  **PKC**  contributes to the mechanism s by which these two kinases regulate neurotransmitter release.  Ref: 10461881 J Neurochem, 1999
  - Serine 842 is phosphorylated by PKA,  **PKC** , and  **CaMKII**  in vitro and is phosphorylated in transfected cells by PKA.  Ref: 10366608 J Neurosci, 1999
  - There was no effect of acidosis on  **PKC**  activity, or on translocation of  **CaMKII**  to cell membranes.  Ref: 10592293 Brain Res, 1999
  - The phosphorylation of the glutamate receptor 1 GluR1 subunit of AMPA receptors by protein kinase A PKA, protein kinase C  **PKC** , and calcium calmodulin dependent protein kinase II  **CaMKII**  has been characterized extensively.  Ref: 10366608 J Neurosci, 1999
  - In the present study, we examined whether the expression of some of the genes for proteins involved in the intracellular calcium signal transduction is also concentrated in the mushroom bodies of the honeybee by isolating cDNA fragments that encode the calcium calmodulin dependent protein kinase II  **CaMKII**  and protein kinase C  **PKC**  homologues of the honeybee.  Ref: 10701869 J Comp Neurol, 2000
  - Furthermore, the enzymatic activities of  **CaMKII**  and  **PKC**  were found to be higher in the mushroom bodies central bodies than in the optic and antennal lobes of the worker bee brain.  Ref: 10701869 J Comp Neurol, 2000
  - The aim of the present study was to investigate the possible causal relationship between protein kinase activity and the induction of the vestibular compensation process, by delivering inhibitors of protein kinase C  **PKC**  or calcium calmodulin dependent kinase II  **CaMKII**  into the ipsilateral VNC at the time of the UVD and determining their effects on three static symptoms of UVD, spontaneous nystagmus SN, yaw head tilt YHT and roll head tilt RHT in guinea pigs.  Ref: 11056183 Brain Res, 2000
  - Sequencing of tryptic phosphopeptides from syntagI revealed that  **CaMKII**  and  **PKC**  phosphorylated the same residue, corresponding to Thr112.  Ref: 10461881 J Neurochem, 1999
